# Supplementary material for: From hospital to home: A heightened window of vulnerability post-critical illness
Source: PLoS One. 2025 Oct 8;20(10):e0334092. doi: 10.1371/journal.pone.0334092 (PMC12507260; doi:10.1371/journal.pone.0334092)
Supplement: S1 File — (DOCX) [file pone.0334092.s001.docx]

**Supporting Information 1**

The file contains 2 interview guides. Interview Guide (i): ICU Survivor-Family Dyads – BASELINE (1 month post-hospital discharge) was used in interviews with patients and their family members who had been discharged home from an initial ICU stay. Interview Guide (ii): ICU Survivor-Family Dyads – HOSPITAL READMISSION was used in interviews with patients and their family members who had been readmitted to hospital following a prior ICU admission.

**Interview Guide (i): ICU Survivor-Family Dyads – BASELINE (1 month post-hospital discharge)**

***Health-priorities***

1. Now that you are home, what is most important to you/family caregiver?
2. Describe your current life priorities and the things you would like to focus on most.
3. Describe the types of activities that are most important to you/family caregiver most?
4. How are your priorities similar or different? (differences between survivor & family)
5. When you think about your health, what worries you the most?

***Health Challenges***

1. Now that you are home, what is most challenging for you/family caregiver?
2. What do you/family caregiver struggle with in your day-to-day life?
   1. Related to your health or well-being (medical and psychological)?
   2. Related to functioning day-to-day (functional)?
   3. Related to the people in your life (relationship)?
   4. Other?
3. Tell me about how your/family caregivers’ roles and responsibilities both in and outside the home have changed from before you were ill.
   1. How has this been for you/family caregiver?
   2. How has your relationship with your caregiver/the person you are providing care for changed?

***Illness Management and Health seeking***

1. What types of things have you/family caregiver tried to do to cope with your ongoing health issues?
2. How have you/family caregiver felt about your need for care and assistance since you’ve been home?
3. In terms of managing your ongoing health issues, what are some of the positives of being at home?
4. Describe any information, instructions, resources or preparation, if any, you received to assist you with your recovery at home.
5. How were these helpful/unhelpful?
6. Describe discussions you have had, if any, with health care providers about your health and recovery?
7. How were these helpful/unhelpful?
8. Describe discussions, if any, you have had with your family/friends about your health and recovery?
9. How were these helpful/unhelpful?
10. Tell me about the people you have interacted with since hospital discharge. Are there people (family, friends, healthcare providers) who have made things easier or harder? Could you provide an example?

***Important Considerations for Post-ICU Care***

1. What could have been done better to help you/family caregiver transition home?
2. Information about financial support, other resources, services, support?
3. What is important for health care providers to consider in creating supports and resources to help ICU survivors like you when they are discharged home?
4. If an ICU follow-up clinic was available to you, what would you like the clinic to help you with?
5. What do you anticipate wanting in the future to help you manage or get help to manage your health?

**Interview Guide (ii): ICU Survivor-Family Dyads – HOSPITAL READMISSION**

***Health Challenges and Hospital Readmission***

1. I understand you became ill enough to need to be readmitted to the hospital. Can you describe the health challenges you experienced when you were at home that led you to needing to go back into hospital?
2. Between the time when you were home and when you went back to the hospital, what was challenging for you/family caregiver?
3. Related to your health or well-being (medical and psychological)?
4. Related to functioning day-to-day (functional)?
5. Related to the people in your life (relationship)?
6. Other?

***Illness Management and Health seeking***

1. What do you think contributed to the decline in your health and hospital readmission?
2. Tell me about how you/family caregiver tried to manage or get help to manage the problems you were having.
3. Tell me about the people you interacted with when you were trying to manage your health at home
4. Were there people (family, friends, healthcare providers) who made things easier or harder? Could you provide an example?
5. Describe discussions, if any, you had had with your family/friends about the problems you were having?
   1. Describe discussions, if any, you had with health care providers about the problems you were having?
6. What, if any, mental health problems have you experienced that you have/ have **NOT** been able to get help for?
7. Please describe the barriers that you have encountered
8. What, if any, physical health problems you experienced that you have/ have **NOT** been able to get help for?
9. Please describe the barriers that you have encountered
10. What has been most difficult for you/family caregiver when you needed more or different medical care?
11. In terms of managing your health problems, what were the positives of being at home?

***Health-priorities***

1. Since being back in and now back out of the hospital, have any of your/family caregivers priorities changed?
2. What is most important to you now?

***Important Considerations for Post-ICU Care***

1. What could have been done better to help your/family caregiver at home?
2. Information about financial support, other resources, services, support?
3. What is important for health care providers to consider in creating supports and resources to help ICU survivors like you when they are at home?
4. If an ICU follow-up clinic was available to you, what would you like the clinic to help you with?
5. What do you anticipate wanting in the future to help you manage or get help to manage your health?
